# Targeting inhaled aerosol delivery to upper airways in children: Insight from computational fluid dynamics (CFD)

**DOI:** 10.1371/journal.pone.0207711

**Published:** 2018-11-20

**Authors:** Prashant Das, Eliram Nof, Israel Amirav, Stavros C. Kassinos, Josué Sznitman

**Affiliations:** 1 Department of Biomedical Engineering, Technion - Israel Institute of Technology, Haifa, Israel; 2 Department of Pediatrics, Faculty of Medicine & Dentistry, University of Alberta, Edmonton, Alberta, Canada; 3 Computational Sciences Laboratory (UCY-CompSci), Department of Mechanical and Manufacturing Engineering, University of Cyprus, Kallipoleos Avenue 75, Nicosia 1678, Cyprus; Coastal Carolina University, UNITED STATES

## Abstract

Despite the prevalence of inhalation therapy in the treatment of pediatric respiratory disorders, most prominently asthma, the fraction of inhaled drugs reaching the lungs for maximal efficacy remains adversely low. By and large drug delivery devices and their inhalation guidelines are typically derived from adult studies with child dosages adapted according to body weight. While it has long been recognized that physiological (e.g. airway sizes, breathing maneuvers) and physical transport (e.g. aerosol dynamics) characteristics are critical in governing deposition outcomes, such knowledge has yet to be extensively adapted to younger populations. Motivated by such shortcomings, the present work leverages in a first step *in silico* computational fluid dynamics (CFD) to explore opportunities for augmenting aerosol deposition in children based on respiratory physiological and physical transport determinants. Using an idealized, anatomically-faithful upper airway geometry, airflow and aerosol motion are simulated as a function of age, spanning a five year old to an adult. Breathing conditions mimic realistic age-specific inhalation maneuvers representative of Dry Powder Inhalers (DPI) and nebulizer inhalation. Our findings point to the existence of a single dimensionless curve governing deposition in the conductive airways via the dimensionless Stokes number (Stk). Most significantly, we uncover the existence of a distinct deposition peak irrespective of age. For the DPI simulations, this peak (∼ 80%) occurs at Stk ≈ 0.06 whereas for nebulizer simulations, the corresponding peak (∼ 45%) occurs in the range of Stk between 0.03-0.04. Such dimensionless findings hence translate to an optimal window of micron-sized aerosols that evolves with age and varies with inhalation device. The existence of such deposition optima advocates revisiting design guidelines for optimizing deposition outcomes in pediatric inhalation therapy.

## Introduction

Inhalation therapy is a hallmark in the treatment of pediatric respiratory disorders, including foremost asthma [1–3]. Not only is asthma the leading chronic disease globally [4], recent studies depict a rise in childhood prevalence over the past decade [5–8]. In this context, inhaled corticosteroids are recognized as effective drugs to suppress airway inflammation [9] and the regular use of such therapeutics at low dose is acknowledged to reduce the risk of morbidity and mortality [10, 11]. The benefits of inhalation therapy include immediate therapeutic action following drug deposition in the tracheobronchial region, lower dosage requirements [12] and reduced side effects when compared to oral administration [2].

In practice, pediatric asthma is commonly treated via the inhalation route [3, 13] using nebulizers, pressurized metered-dose inhalers (pMDI) or dry powder inhalers (DPI). While each device family holds its own merits and limitations in delivering therapeutics to a child’s respiratory tract [14], nebulizers and MDIs are widely recommended in younger pediatric populations [1, 15], i.e. less than 5-6 years of age. This follows as nebulizers and MDIs are considered active devices since they generate aerosols independent of a patient’s effort. In contrast, DPIs, which require patient compliance via quick and vigorous suction to de-aggregate the drug powder [16], are advocated in older children who can successfully execute such maneuvers. Despite the prevalence of pediatric inhalation therapy, the fraction of inhaled drugs reaching the lungs for maximal therapeutic efficacy is still critically low in children [17, 18]. Following administration, much of the inhaled dose is typically lost in the extra-thoracic regions (e.g. mouth-throat). Past *in vivo* studies report deposition efficiencies typically ranging between 0.5% and 12% for nebulizers [17], whereas DPIs and pMDIs yield efficiencies nearing 40% in the best of cases [17–19]. Such shortfalls call for increased efforts in achieving improved, if not optimal, therapeutic delivery in pediatric populations [20].

While behavioral and emotional aspects unique to children [21, 22] as well as poor adherence to treatment [23, 24] carry adverse effects on disease control and treatment, the specifics of childhood lung physiology (e.g. airway sizes, breathing maneuvers, etc.) in conjunction with aerodynamic (e.g. aerosol transport) factors are critical in governing aerosol deposition outcomes [25]. Yet, such physiological and physical characteristics are still widely overlooked: established drug delivery devices and their inhalation guidelines are commonly derived from adult studies [21, 26–28], whereby pediatric dosages, that is the amount of drug prescribed (e.g. mg) over a given amount of time (e.g. every 12h) for a specific therapy (e.g. bronchodilators), are adapted according to body weight [29, 30]. Nonetheless, seminal *in vivo* studies in adults have long acknowledged the critical role of particle size in determining pulmonary deposition and thus deposition efficiency [31–33]. Such knowledge has yet to be extensively explored in, and adapted for, younger populations. Concurrently, age-related changes in lung morphology need to be considered: as the lungs mature through childhood, the conducting airways grow in dimension and tidal volumes increase with age up until adulthood [34–36]. Such anatomical transformations occur in parallel with changes in breathing frequency [37] and influence deposition outcomes [15] that differ significantly between children and adults [21].

Over the past decades, computational fluid dynamics (CFD) have proven valuable in investigating pulmonary flow phenomena and predicting the fate of inhaled pharmaceutical aerosols with reasonable fidelity [38–41]. In particular, *in silico* approaches offer the opportunity to quantitatively map the physical flow and particle transport determinants underpinning pulmonary deposition outcomes. Yet, the vast majority of *in silico* studies to date have overwhelmingly focused on adult airways, by relying on idealized airway geometries [42, 43] or patient-specific reconstructed lung anatomies acquired from imaging modalities (e.g. computed tomography) [44]. This follows in the absence of widely-available imaging (e.g. CT) or deposition (e.g. gamma-scintigraphy) data in children, owing in part to ethical concerns [45]. Age-dependent studies have in turn been traditionally limited to theoretical models [46–48]. More recently, CFD studies have begun exploring child-specific respiratory flow patterns [49] and particle deposition outcomes in idealized upper-airway models [50] and the deep pulmonary acinar regions [51], including in childhood asthma [52]. Despite such progress, pediatric *in silico* studies have mostly considered airway models distal to the trachea, thereby neglecting the mouth-throat region’s role as a filter during inhalation [53, 54]; a significant concern in predicting the effective dose delivered in children [55, 56]. Moreover, studies have mostly ignored the influence of inhalation maneuvers in children (e.g. rapid inhalation with DPI) on lung deposition outcomes.

Motivated by ongoing shortcomings in childhood inhalation therapy, we leverage in the present work *in silico* CFD methods to explore opportunities for augmenting aerosol deposition based on respiratory physiological and physical transport determinants specific to pediatric populations. To this end, our *in silico* efforts focus in a first step on aerosol deposition outcomes in an anatomically-realistic lung model for three age points, ranging from a five year old child to an adult. The age-specific upper airway models comprise the mouth-throat region, trachea and conductive region spanning a total of seven asymmetric generations. For each age, we investigate two distinct breathing maneuvers relevant for DPI and nebulizer inhalation. Upon characterization of the ensuing flow patterns, we systematically map the deposition characteristics for a broad range of aerosol sizes relevant to the two inhalation devices. Our numerical simulations exemplify the evolving relationship between aerosol deposition characteristics, particle size, and age. Our findings support the prospect of child-specific inhalation therapies driven by selecting aerosol size for optimal deposition in the upper tracheo-bronchial airways.

## Methods

### Airway geometry

**Fig 1:**
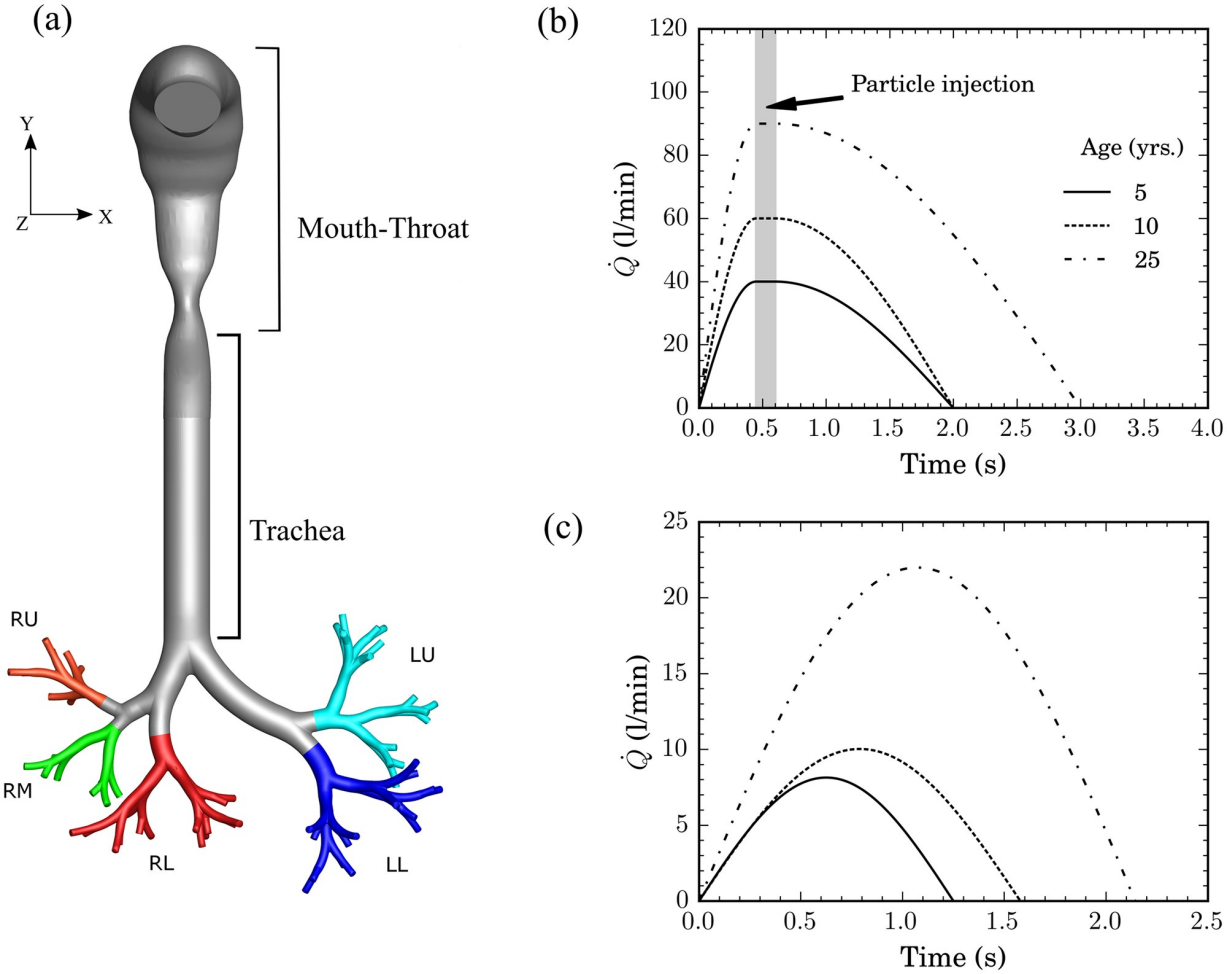
(a) Computer-aided design (CAD) of the respiratory airway geometry including mouth-throat, trachea and conducting airways (7 generations). The different branches leading to the distinct lobes of the lungs are color-coded (i.e. RU- Right Upper, RM—Right Middle, RL—Right Lower, LL—Left Lower, LU—Left Upper). The geometry is scaled self-similarly according to selected age points (i.e. 5, 10 and 25 years old). Profiles of the inhalation maneuvers (i.e. flow rates) as a function of time are shown for (b) Dry Powder Inhaler (DPI) and (c) nebulizer simulations, respectively. For each inhalation maneuver, the three different age points are independently investigated. Particle injection in DPI simulations is confined to a short bolus, spanning 0.45 s to 0.6 s, for all age points (see shaded region in (b)). For nebulizer simulations, aerosols are injected continuously throughout the inhalation period.

We simulate the transport and deposition of therapeutic aerosols under realistic DPI- and nebulizer-like inhalation profiles. The geometries consist of an idealized, morphometrically-realistic upper airway model of the human lung that varies as a function of age, featuring a mouth-throat geometry coupled with a trachea-bronchial tree. The extra-thoracic (i.e. mouth-throat) region follows an idealized model introduced and validated by Xi & Longest [53] and made available through RDD Online [57]. In parallel, the conductive bronchial airway tree features a morphometrically-faithful model of an average human (male) adult [58]. Briefly, the airway tree features seven generations of dichotomously branching, asymmetric airways that follows the seminal morphometric models of Weibel [59] and Horsfield [60], using previously reported methods [61]. The 3D computer-aided drawing (CAD) geometry is readily made available in the Supporting Information (S1 File). The complete airway geometry is illustrated in Fig 1a. Note that, following respiratory physiology (see details below), the 64 distal outlets are grouped by their respective downstream region of the lungs (i.e. Right Upper Lobe, Right Middle Lobe, Left Upper Lobe, etc.) and color-coded for clarity in Fig 1a. To quantify subsequent deposition characteristics (see Results and discussion), the complete geometry is divided into three regions of interest: the mouth-throat region, the trachea and all remaining conducting airways.

To mimic representative airways of pediatric populations at a given age point, the adult geometry is homothetically scaled down. Previous studies have shown that to a first approximation the upper airways in children and adults exhibit geometrically similar features [55]. This has been further corroborated in experimental studies which show that a uniform down-scaling of idealized adult airways yields reasonably accurate aerosol deposition estimates in pediatric airways [55, 62]. We note that in these referenced studies, the best-fit of the scaling factors found for scaling down the mouth-throat (i.e. scaling factor of 0.62 for the broad age groups ranging between 6–14 years) and the conducting airways (i.e. scaling factor of 0.56 for age groups 4-8), respectively, are slightly different from one-another when adapting the anatomies from adulthood to childhood. Note that differences between these two factors may not necessarily be a reflection of intrinsic differences in anatomical growth with age for the different lung regions but may also lie in intra-species differences from the specific adult model which each study is initially based on. In the present study, the scaling factors are selected to match the representative tracheal diameter at each select age point relative to the adult geometry (i.e. 25 year old) [35, 36]. This yields a homothetic factor of 0.578 and 0.743 at 5 and 10 years old, respectively. Representative anatomical airway dimensions are summarized in Table 1 according to age. Note that the scaling factor at 5 year old falls well within the aforementioned numbers [55, 62] and thus yields airway geometries that lie within the physiological range. We recall that the present study is not intended to explore interspecies differences (e.g. patient-specific characteristics) or anatomical deviations from the baseline airway geometry (e.g. in case of airway dysanapsis [63]). Moreover, our work is limited to assessing deposition in the upper conductive airways and thus neglects the fate of inhaled aerosols reaching more distally into smaller bronchi and beyond. Nevertheless, our canonical approach serves the purpose to characterize as a first step the underlying similarities and differences in aerosol transport that are uniquely bound to the intrinsic scales of the upper airway anatomy.

**Table 1:** Selected airway diameters as a function of age. Note that for the mouth, the reported values correspond to the hydraulic diameter.

Diameter (mm)	Age (yrs.)		
	5	10	25
Mouth	11	14	19
Trachea	9	11.6	16
Bronchi (Left & Right)	6.2	8	10.8

### Inhalation maneuvers

The representative breathing maneuvers used to simulate DPI and nebulizer inhalation profiles are shown in Fig 1b and 1c, respectively, for all age groups. The time-dependent inhalation profile for a DPI has been previously reported in the literature [64] and explored in adults [65]. We have modified this original profile for the chosen pediatric age points (Fig 1b) to reflect changes in total volume inspired and inhalation duration [37, 66, 67]. The inhalation parameters such as tidal volume, peak inspiratory flow rate (PIFR) and the duration of inspiration (*t*_*insp*_) are summarized in Table 2. Fig 1c shows the nebulizer inhalation maneuvers which are typically modeled with sinusoidal functions representing tidal breathing [68, 69]. By implementing the relevant breathing frequency [37] and tidal volume [35], we reconstruct inhalation profiles for the three age points studied (see Table 2). Note that in the present study the youngest age point explored is 5 years old, as younger populations cannot typically operate DPIs [1, 15].

**Table 2:** Selected inhalation parameters as a function of age and breathing maneuver.

Age (yrs.)	DPI			Nebulizer		
	5	10	25	5	10	25
Tidal Volume (l)	0.89	1.33	2.95	0.108	0.168	0.5
PIFR (l/min)	40	60	90	8.14	10	22
*t*_*insp*_(s)	2	2	3	1.25	1.58	2.14

### Numerical flow simulations

Time-dependent airflow simulations are carried out using a commercial software (Fluent 18.2, ANSYS, Inc.), whereby the mass and momentum (i.e. Navier-Stokes) equations are solved numerically. Most unsteady respiratory flows typically involve laminar, transitional, and turbulent flow regimes [40, 70], in particular in the extra-thoracic regions where instantaneous local Reynolds numbers (Re) can range between a few hundred to values exceeding 5’000 during heavy breathing. For such flow scenarios, the *k*-*ω*-SST (Shear Stress Transport) model has been shown to be reasonably accurate in simulating the relevant airflows [40, 69], also taking into account low-Re corrections. In this model, the transport equations for turbulent kinetic energy (*k*) and specific dissipation rate (*ω*) are solved in addition to the mass and momentum equations. In the present study, we implement the *k*-*ω*-SST turbulence model with second-order upwind schemes for *k*, *ω* and momentum. A second-order implicit scheme is used for the transient formulation, with a time step of 10^−3^ s (≪ *t*_*insp*_) to ensure good accuracy in resolving the unsteady flows over the relevant inhalation cycle.

For numerical modeling purposes, the mouth opening is treated as a velocity inlet to conform to the desired time-dependent inhalation flow rates (Fig 1). In parallel, the boundaries of the airways are treated as a wall, with a no-slip condition implemented for airflow. Here, the flow outlet conditions are chosen to approximate realistic flow distribution to each lung lobe of the airway geometry; a computational approach in line with recent works [71]. Namely, the distal branches (i.e. 64 outlets) are grouped into five lobar regions (Fig 1a), thereby approximating outflow into the five lobes of the lungs. Outflow conditions are weighted to ensure that mass flow distribution through each lobe are in line with established physiological estimates [71–73]; namely 15% to the left upper (LU) lobe, 31% to the left lower (LL) lobe, 14% to the right upper (RU) lobe, 7% to the right middle (RM) lobe and 33% to the right lower (RL) lobe. To this end, the fractional flow rate boundary condition option is implemented in ANSYS Fluent. Briefly, this signifies that pressure at the outlets is not directly specified but rather, values are determined by the solver using the upstream flow conditions. Here, the “flow rate weighting” option is used to specify what percentage of the inlet flow is distributed to each lung lobe.

The airway geometry was discretized using tetrahedral elements in ICEM (ANSYS Inc., Canonsburg, PA) with prism layers at the airway walls. The resulting mesh was imported into Fluent and converted into a polyhedral mesh, the benefits of which for simulating respiratory flows have been recently discussed [74]. Rigorous mesh convergence tests were performed (i.e. ranging from 2M to 6M tetrahedral cells) to eventually select the final mesh of ∼900,000 polyhedral cells (converted from ∼2.4M tetrahedral cells), with up to 10 prism layers for near-wall refinements. Refinements based on curvature and airway branch size were also included to deliver a high-quality mesh. Briefly, centerline velocity profiles and secondary flow streamlines at selected locations near the trachea and bronchi were compared with the finest mesh size to ensure that the maximum variations in the selected mesh were < 2% of the corresponding values. To study different age points, the mesh was scaled self-similarly following the scaling factors defined above.

### Aerosol transport and deposition

To simulate the inhalation of an aerosol bolus, particles are released at the mouth inlet and tracked throughout the domain. The release time of aerosols is usually chosen such that it reasonably approximates the true release from an inhalation device. For DPI, particle release is typically implemented in the first 0.5 s of the inspiratory flow [75], corresponding to the approximate duration for the device to empty. However, the true release time and duration are acknowledged to be dependent on various parameters including inhalation flow rate, inhalation profile and the design of the device itself [75]. Moreover, the release of particles from the device is known to take a finite amount of time after the onset of inhalation and is thereby dependent on inhalation flow rate [76]. Within the scope of the present work, we have chosen to minimize the additional variability in our problem definition by selecting a constant particle release time across the three age points studied. We have thus selected the peak inspiratory flow rate (i.e. PIFR) under DPI inhalation as the starting point for particle release. Note that in our models, PIFR is assumed to be reached at 0.45 s for the three age groups. Particles are released continuously during PIFR, which lasts until 0.6 s. Although this modeling approach produces a relatively short bolus release, it facilitates the comparison of different age points in terms of deposition efficiency. For the nebulizer studies, particles are continuously injected during the entire inhalation phase.

For all inhalations scenarios, we implement a uniform injection of particles at the mouth inlet with a total of approximately 28,500 particles of each size of interest to ensure good deposition statistics [71, 77]. This translates to a total of 342,000 particles in DPI simulations and 570,000 particles in nebulizer simulations respectively. We track spherical particles (i.e. particle density *ρ*_*p*_ = 1000 kg/m^3^) with diameters spanning 1-12 *μ*m (for DPI inhalation maneuvers) and 1-20 *μ*m (for tidal breathing) [71, 78]. Since particle concentrations in the air-phase are typically very low, we employ a Lagrangian based, one-way coupled, discrete phase model to simulate particle motion [79]. The main forces governing the transport and deposition of particles are assumed to be viscous drag (i.e. Stokes drag for small particles) and gravitational sedimentation [80] (note that gravity is in the negative Y direction, Fig 1a). For particles with ≥ 1 *μ*m, the stochastic force resulting from Brownian motion may be neglected [81]. Note that other effects related to electrostatic charge or hygroscopic growth are not explored here; a limitation that lies beyond the scope of the present study [82]. Finally, particles impacting on the airway walls are assumed to have deposited. For each inhalation scenario (e.g. DPI at 5 years old), a minimum of three independent simulations were executed to ensure repeatability of deposition statistics. The maximum discrepancy in deposition efficiency outcomes was found to be < 1% between runs for any given inhalation scenario.

## Results and discussion

### Flow characteristics

**Fig 2:**
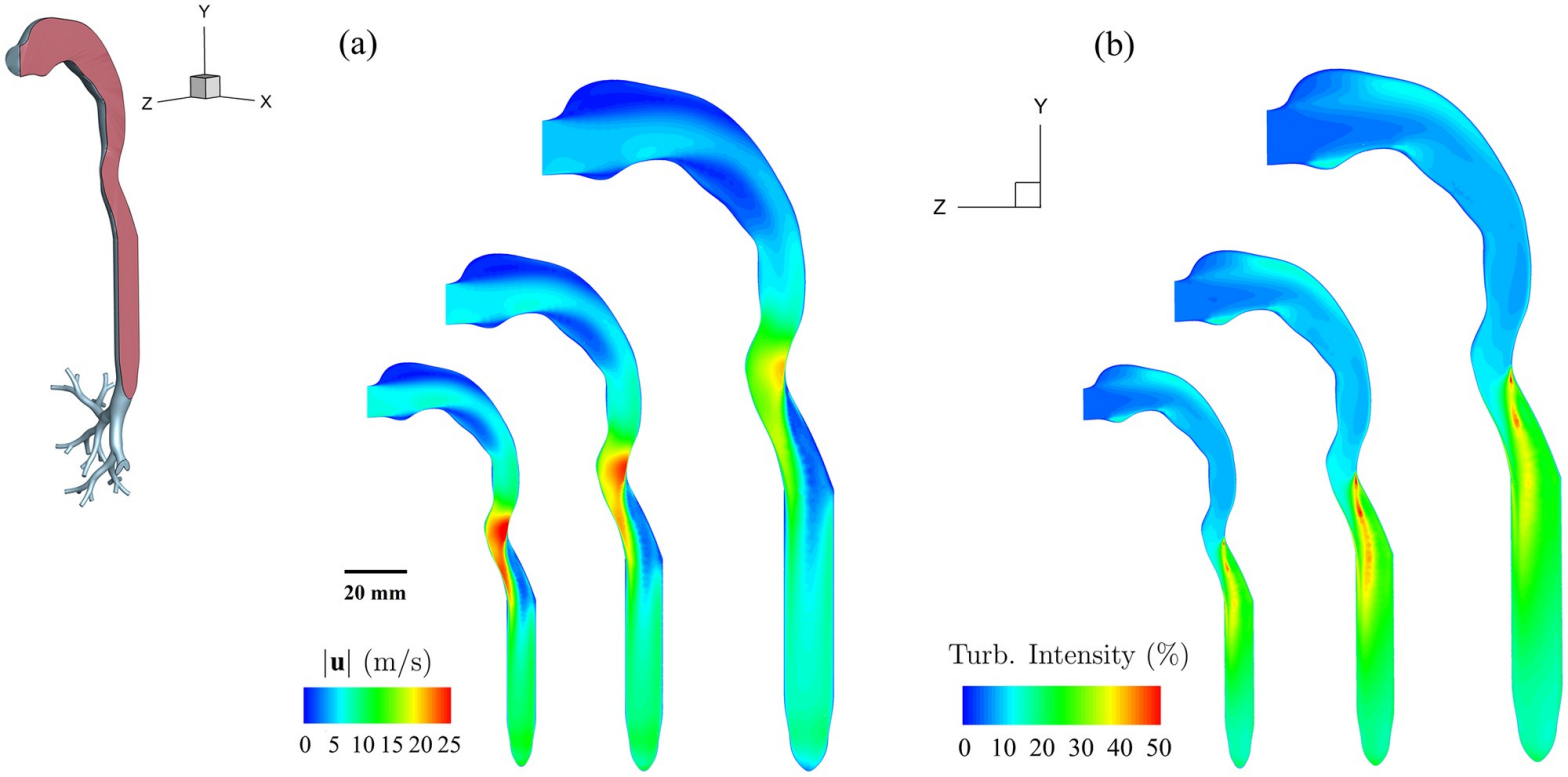
Contour maps of (a) mean velocity magnitude (|u|) and (b) turbulent intensity at peak inhalation (i.e. 0.45 s) for DPI simulations (see inhalation profile in Fig 1b). Contour maps are rendered across the center plane cutting through the mouth-throat and trachea; the 2D center plane is schematically shown in the sliced section of the 3D CAD geometry on the left. Contours are shown across the three age points of 5, 10 and 25 years, corresponding to the geometries of increasing size in (a) and (b), respectively.

To gain initial insight into the underlying respiratory flow characteristics as a function of age, Fig 2a shows contours of the velocity magnitude (|**u**|) for a DPI inhalation maneuver. Note that the instantaneous flow fields are shown at their peak strength, when inhalation reaches PIFR at 0.45 s (see Fig 1b). Results are shown along the centerline plane running through the mouth-throat and the trachea for the three distinct age groups. The instantaneous Reynolds numbers at the mouth inlet are approximately 5’000, 5’800 and 6’500 for the 5, 10 and 25 years old cases, respectively. While PIFR increases with age (both in DPI and nebulizer; Table 2), the mouth inlet area increases as well (Table 1) such that we observe comparatively larger inlet flow velocities for the younger ages (Fig 2a). We note that the velocity magnitude contours look qualitatively similar for the three age groups, as anticipated for the range of similar peak Re at the mouth. A notable signature of the flow is the formation of a laryngeal jet in the presence of the larynx constriction; a feature previously discussed [40, 83]. Numerical simulations have shown that the larynx is an important anatomical element that influences aerosol deposition outcomes [84]: flow acceleration due to the constriction coupled with directional changes due to anatomy contribute to enhanced aerosol deposition near the larynx [83]. Here, the laryngeal jet is observed across all age points at PIFR, with a peak velocity magnitude reaching a local maximum in the vicinity of the larynx contraction. Some apparent differences arise nevertheless with respect to age. The local flow velocity magnitudes increase for younger ages due to the smaller airway diameter of the constriction. In turn, values can reach as high as 25 m/s for the 5 year old model during PIFR. Under nebulizer inhalation (see S1a Fig), flow patterns across different age points are seen to exhibit similar qualitative features as those for DPI. However, since the inhalation flow rates are comparatively weaker (compare Fig 1b and 1c), the local peak flow velocities near the larynx yield correspondingly smaller magnitudes (e.g. 6 m/s at PIFR at 5 years old, S1a Fig).

Fig 2b shows the corresponding contour maps of the instantaneous turbulent intensity at PIFR across the center plane for all three cases using DPI. The turbulence intensity is defined as the ratio between the root-mean-square velocity fluctuations and a reference flow velocity (i.e. chosen as the instantaneous inlet velocity at the mouth). It can be noted from the turbulent intensity contours that in all cases the instantaneous value is found to be highest in the vicinity downstream of the laryngeal jet, i.e. within the trachea. This is anticipated from the presence of large velocity gradients (i.e. high shear), as seen from Fig 2a. As one moves more distally down the airway tree, turbulence intensity values become more uniform, i.e. on the order of 20-30% during PIFR for all cases. The intensity of turbulence in the tracheal region of adults has been reported to reach up to 20% in previous numerical studies [85], even for peak flow rates as low as 20 l/min. In the distal branches, as the effects of the laryngeal jet subside, turbulence intensity reduces to < 10% even at PIFR. With the decrease in flow rate past PIFR, the turbulence intensity begins to gradually decrease. However, since in DPI simulations the overwhelming majority of inhaled aerosols either deposit or exit the distal branches in a relatively short time (within < 0.5 s after release), the flow characteristics at later time instances do not influence significantly aerosol deposition outcomes. It can be noted that in the case of nebulizer inhalation (see S1b Fig), turbulence intensity remains below 10% during PIFR, even near the larynx contraction; values significantly lower than for DPI.

**Fig 3:**
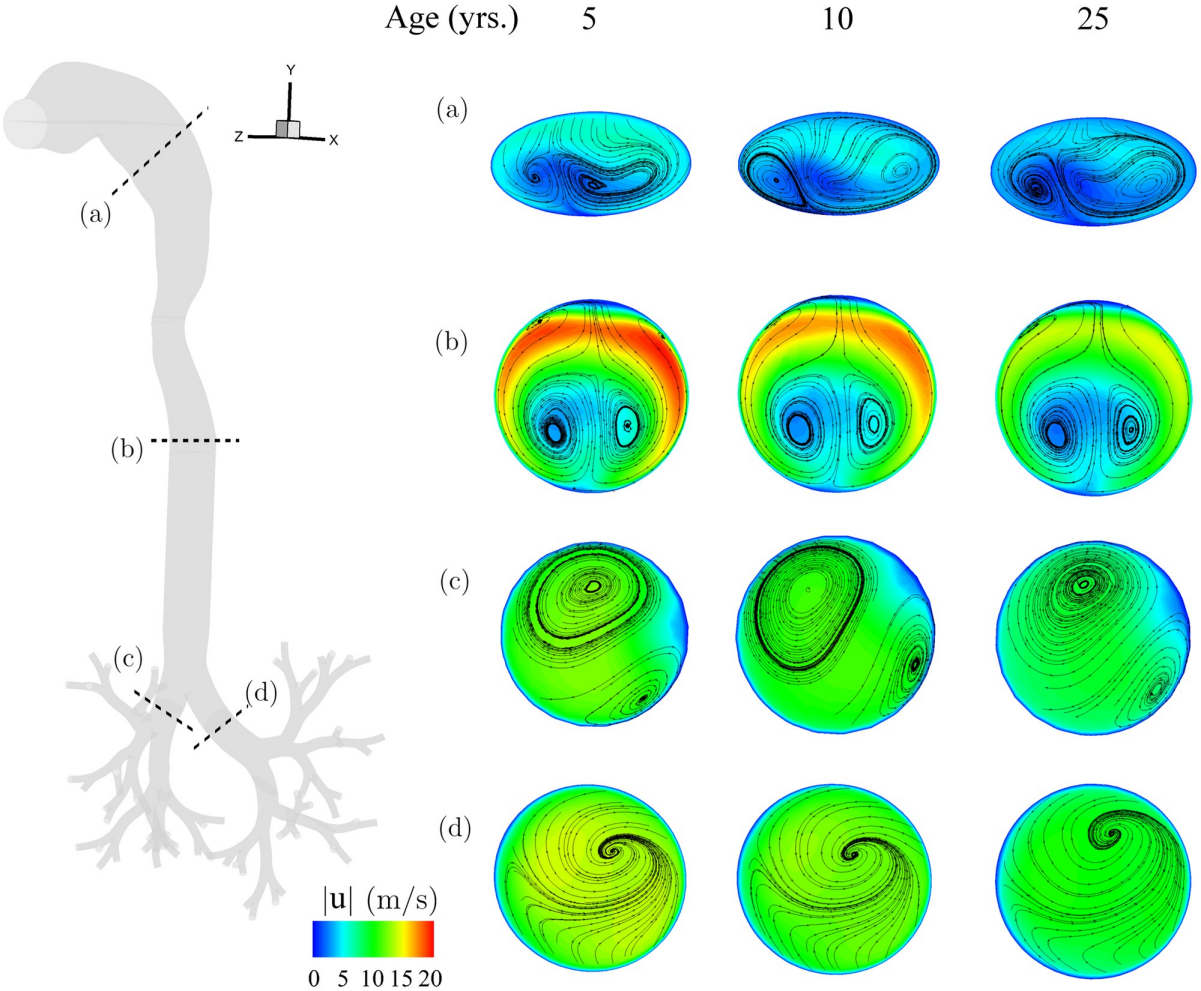
Contour maps of the velocity magnitude (|u|) rendered along with instantaneous secondary velocity streamlines for (a-d) selected cross-sections across the airway geometry and the three age points (columns), respectively. Results are shown for the DPI inhalation maneuver at peak inhalation flow rate (i.e. 0.45 s), as shown in Fig 1b.

To further compare the evolution of respiratory flow fields with age, we briefly consider the instantaneous velocity magnitude contours at different cross-sections along the airway geometry (see Fig 3). Here, cross-sections at all age points are visually scaled to the same size for ease of comparison. In conjunction, the instantaneous streamlines capturing the secondary flow patterns are shown on each cross-section. At each airway location selected, cross-sectional flow patterns are qualitatively similar, independent of age. Not surprisingly, the secondary flow pattern seen in the mouth-throat region (panel a) resembles that observed by Xi & Longest [53], recalling that we have employed the same idealized mouth-throat geometry. The secondary flow structures near the trachea (panel b) are largely symmetric about the central plane, owing to the symmetry of the mouth-throat and tracheal region. However, past the bifurcation at the carina (as the airway splits asymmetrically into the right and left lung), asymmetrical secondary flow patterns arise in the main bronchi. This asymmetry in secondary flows is carried across all age points. In more distal airway branches (not shown for brevity), however, secondary flow structures dissipate as inertial effects (i.e. Reynolds number) decrease [40]. Flow patterns return to more quasi-steady parabolic-like profiles even at PIFR in DPI inhalation. The qualitative flow features described here for DPI are also present for nebulizer inhalation, with differences manifesting primarily in the velocity magnitudes (not shown for brevity).

As a final remark, we note that secondary flows are not only associated with location along the airway tree but are also time-dependent due to the transient nature of the inhalation cycle (i.e. when local instantaneous Dean numbers are larger than unity [86]). In the discussion that follows on the fate of inhaled aerosols we recall that particle transport in DPI is bound to a confined aerosol bolus that rapidly transits through the upper airways during a short time window spanning PIFR (about 0.15 s). Deposition is thus anticipated to occur not only rapidly, but also confined within regions of relatively high velocities in the presence of secondary flows. In contrast, nebulized aerosols are administered continuously during the inhalation cycle such that deposition ensues even when secondary flows have subsided across the upper airway tree.

### Particle deposition efficiency

We begin by quantifying deposition efficiency for each inhalation profile (i.e. DPI vs. nebulizer) and respective age group (i.e. 5, 10 and 25 years old). Accompanying SI videos (S1 and S2 Videos) are provided as examples of transient particle dynamics and ensuing deposition patterns that occur during inhalation. Here, deposition efficiency is defined as the ratio of the number of aerosols deposited to the total number of those inhaled. Recalling that for the range of inhalation flow rates involved (in particular for DPI) inertial impaction is anticipated to be a significant factor governing deposition in the oropharyngeal region [87], wherein the aerosol dynamics are typically characterized by the Stokes number. This non-dimensional parameter, defined as the ratio of a characteristic particle time scale to a characteristic flow time scale, quantifies the relative importance of particle inertia in a flow and may be defined as [79, 88]:

$$\begin{array}{c} \text{Stk}=\frac{\rho_{p}d_{p}^{2}U_{m}C_{c}}{18\mu D_{o}}, \end{array}$$ (1)

where *ρ*_*p*_ is the particle density, *d*_*p*_ is the particle diameter, *U*_*m*_ is a characteristic velocity of the flow, *C*_*c*_ is the Cunningham slip correction factor, *μ* is the dynamic viscosity of the fluid (i.e. air) and *D*_*o*_ is a characteristic length scale of the flow (e.g. airway diameter). Here, we take the corresponding hydraulic diameter at the mouth inlet as the characteristic diameter for all cases studied (Table 1). In DPI simulations, we use the average inlet inspiratory velocity during PIFR (i.e. the window of time during which particle injection occurs, Fig 1b) to be the characteristic velocity (*U*_*m*_). For nebulizer simulations, *U*_*m*_ corresponds rather to the mean inhalation velocity taken over the period of inspiration (Fig 1c), as aerosols are released during the entire inhalation duration.

**Fig 4:**
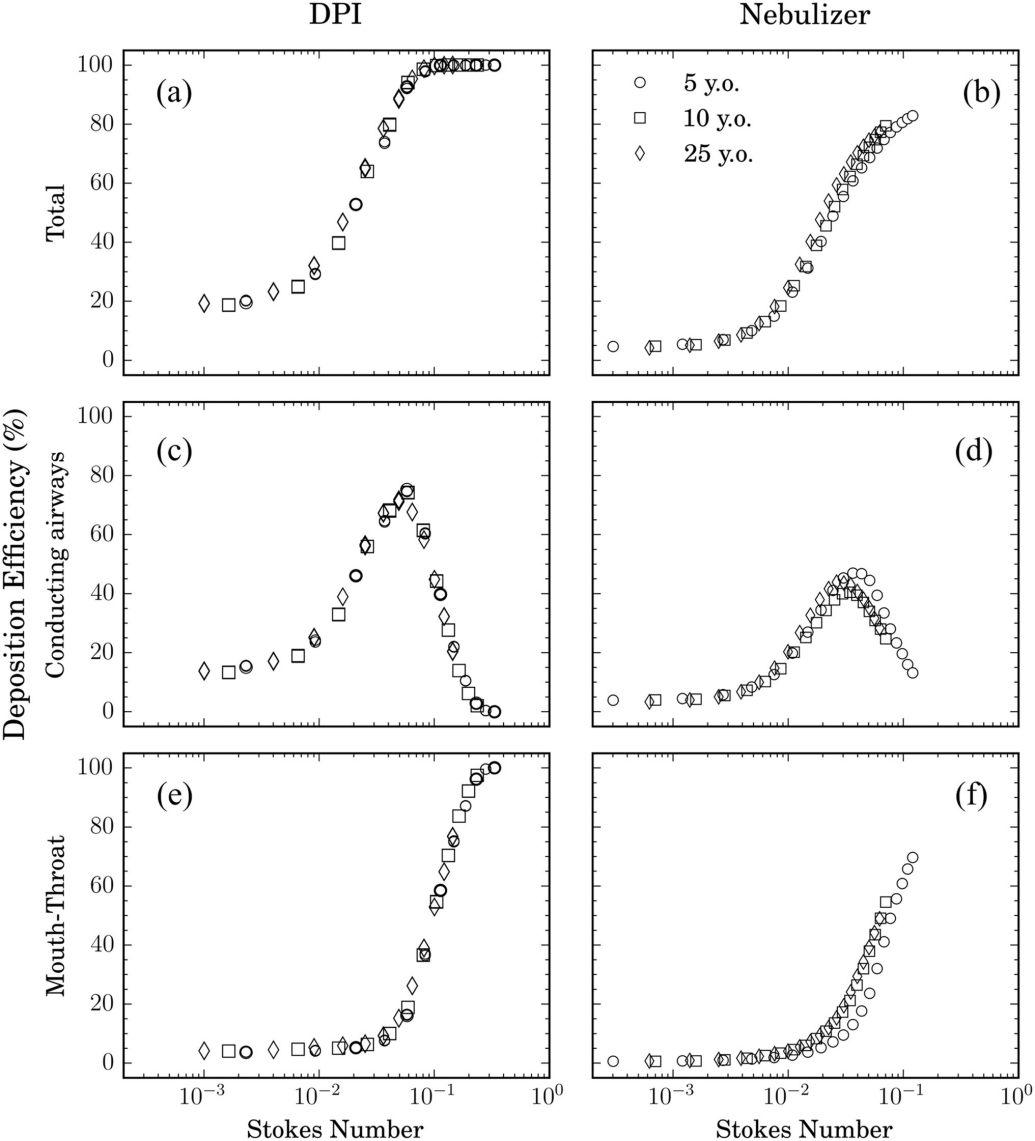
Deposition efficiency as a function of Stokes number for (a, c, e) DPI and (b, d, f) nebulizer simulations, respectively. Results are shown for (a, b) total and (c, d) conductive airway and (e, f) mouth-throat deposition across the three age points investigated.

Fig 4 summarizes resulting deposition efficiencies as a function of Stk across all cases. For each inhalation maneuver we present (i) total deposition efficiency (a & b), (ii) deposition efficiency confined to the conductive airways (c & d) and (iii) mouth-throat deposition efficiency. Within the scope of this work, we have not explicitly monitored particle deposition in each lung lobe, i.e. that is the number (or ratio) of total particles that bypasses our geometry to exit through the various airway outlets and whose deposition fate lies in the respective lung lobes. Indeed, the present efforts focus on assessing deposition fraction outcomes pertinent to upper airways, that include specifically the mouth-throat region, trachea and the conducting airways (as marked in Fig 1a).

Focusing our attention first on the DPI cases, we observe that results across all age points fall on a single curve as a function of Stk (Fig 4a); correspondingly, the deposition efficiency in the mouth-throat region follows a similar trend (Fig 4e). We note that for larger values of the Stokes number (i.e. Stk> 0.1, see Fig 4a), deposition efficiency rapidly yields unity. This results from the relative importance of particle inertia, whereby particles deposit overwhelmingly due to impaction. Similar trends have been reported for oral [78, 89] as well as total (i.e. oral and conducting airways) deposition efficiency in adult upper airways [90]. In particular, Cheng *et al*. [78, 89] have shown *in vitro* for measurements at different steady inspiratory rates and particle sizes that oral deposition efficiency falls on a single sigmoidal curve as a function of Stk, similar to Fig 4a. In parallel, previous deposition studies in nasal airways [91, 92] yield analogous trends, including in infants [89].

Our results emphasize that we recover comparable trends for DPI simulations involving more realistic, unsteady (and age-specific) inhalation profiles where aerosols are released as a short bolus during PIFR. Fig 4b & 4f show the corresponding total and mouth-throat deposition efficiency respectively, during nebulizer inhalation. As in the DPI case, data points fall on a single sigmoidal curve governed by Stk, irrespective of age. In contrast to the DPI case, however, the total and the mouth-throat deposition efficiencies do not reach unity as particles are continuously released throughout the inhalation cycle. These findings (DPI and nebulizer) underscore the relative importance of intrinsic aerosol dynamics (i.e. particle inertia via Stk) relative to other parameters (e.g. inhalation profile, anatomy) in capturing reasonably well the overall deposition characteristics of inhaled aerosols.

We next present in Fig 4c and 4d for the DPI and nebulizer cases, respectively, deposition efficiencies confined to the conducting airway region only; we recall that such deposition outcomes are of specific interest in the context of inhaled therapeutic delivery. Interestingly, we observe that deposition efficiencies fall on a single curve across all age points as a function of Stk, with the emergence of an apparent deposition peak. The general Gaussian-like nature of these curves (with *p* > 0.8 following a Kolmogorov-Smirnov test for all curves) resembles previous thoracic deposition data in adult airways [91]. While Fig 4 underlines how deposition efficiencies follow trends independent of age, the specific aerosol delivery method (i.e. DPI or nebulizer) alters quantitative deposition outcomes. In the DPI case (Fig 4c), the peak in deposition efficiency falls in the vicinity of Stk ≈ 0.06, where nearly 80% of the total inhaled particles are deposited in the conducting airways. For nebulizer inhalation (Fig 4d), this peak is much lower with a maximum of ∼ 45% for Stk in the range of 0.03-0.04. From an aerosol drug delivery perspective, our results point to the fact that in striving for efficient delivery of inhaled therapeutics to the upper conductive airways, a common optimization design road-map would exist, irrespective of age. Indeed, aerosols sizes should be selected according to a specific Stk range for each delivery device; a point we return to in our discussion below.

**Fig 5:**
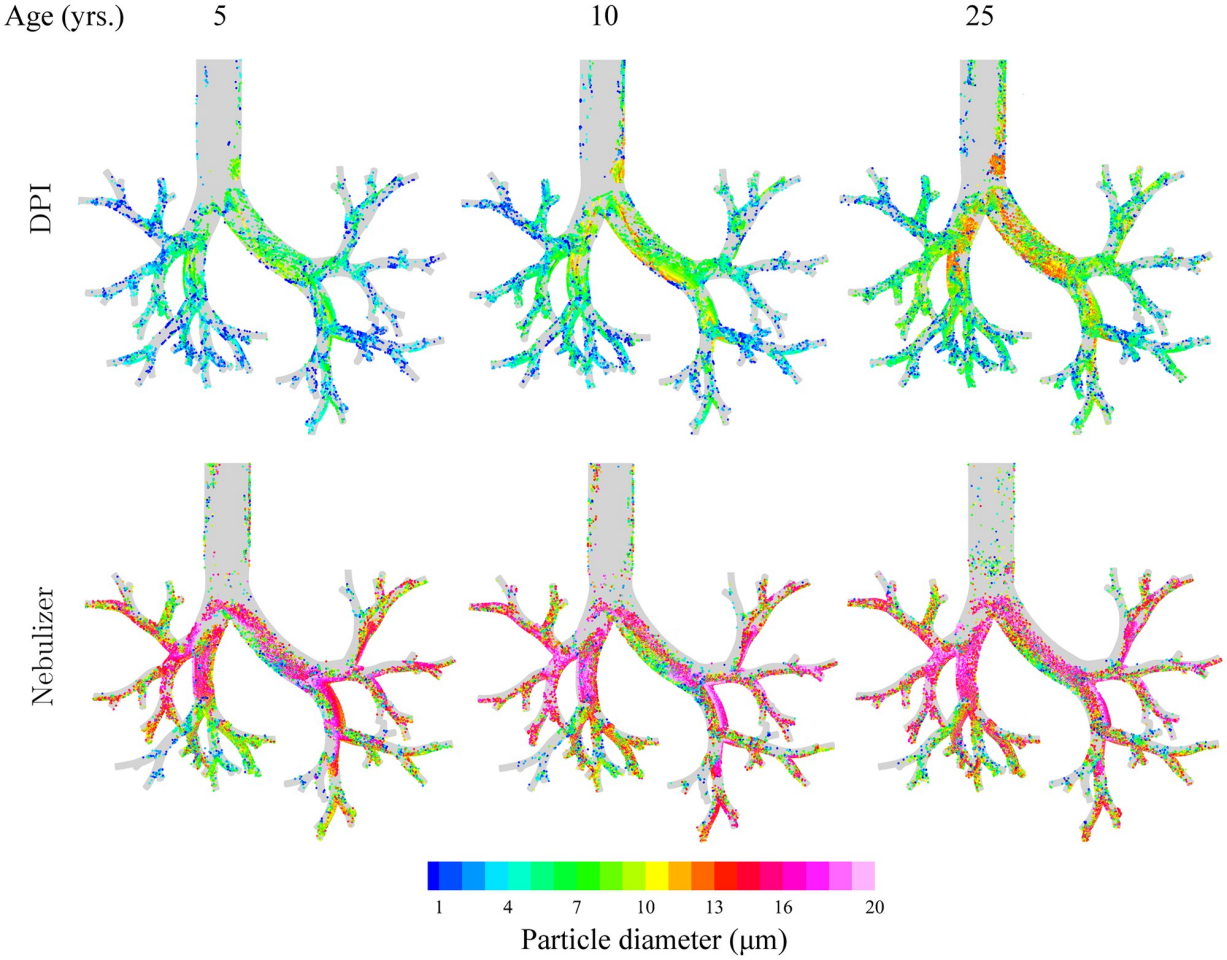
Particle deposition maps in the trachea and conducting airways according to the three age groups. Results are shown for DPI and nebulizer simulations, respectively. Deposited particles are color-coded according to particle diameter (see legend). Note that all airway geometries have been visually scaled to the same size for ease of comparison between age points. Accompanying SI videos (S1 and S2 Videos) provide a visual example of aerosol dynamics and ensuing deposition during inhalation (for DPI in a 5 year old).

In an effort to translate the above deposition results more visually, Fig 5 shows qualitative maps of deposited aerosols in the conducting airways (color-coded according to aerosol diameter). Airway geometries are scaled to the same size for ease of comparison between age points. For each inhalation maneuver (rows of Fig 5), the findings of Fig 4 are spatially highlighted: particle sizes depositing in the conducting airways increase with age, whereby the inhalation maneuver favors the use of larger aerosols for nebulizers compared to DPI; a result captured from the range of Stk values noted earlier in Fig 4c and 4d. Recalling S2 Video, the transient visualization emphasizes how larger aerosols are swiftly deposited in the extra-thoracic region after being inhaled (aerosols with *d*_*p*_ > 6 *μ*m are shown in red). In contrast, smaller aerosols deposit more distally in the conducting airways. With such dynamic insight, we qualitatively note from Fig 5 that for DPI, aerosols smaller than about 4 *μ*m (for the 5 year old) deposit preferentially in the distal airways; a broad feature common across all age groups. In the bronchial region near the carina, deposited particles are instead within a larger size range, beyond 5 *μ*m.

### Optimal aerosol size for pediatric upper airway targeting

**Fig 6:**
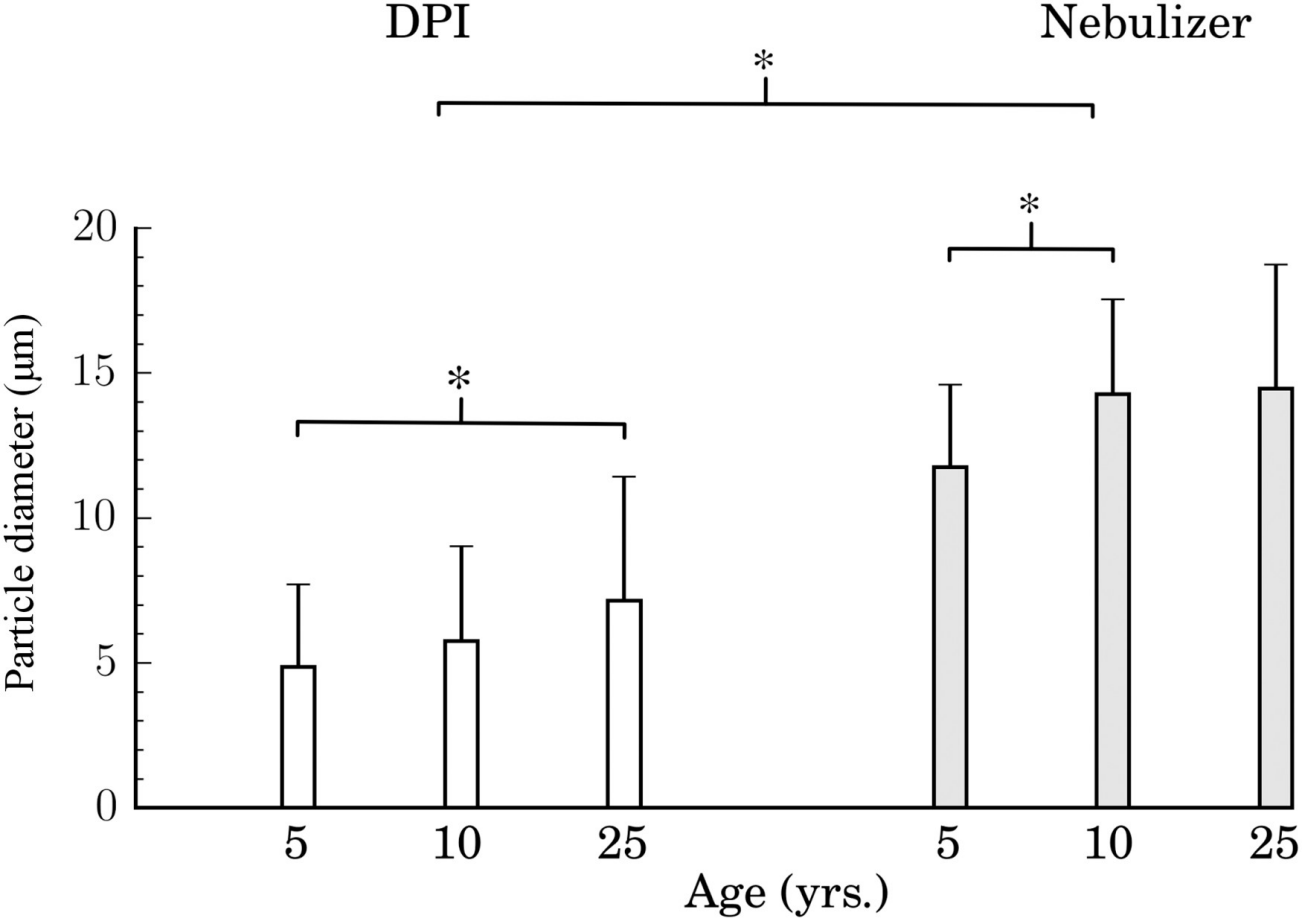
Size distribution of particle diameters for optimized delivery in the conducting airways, as a function of select age points. Results are shown for DPI and nebulizer inhalation maneuvers, respectively. Data are directly extracted by fitting Gaussian curves to the deposition efficiency profiles as a function of Stk (Fig 4c and 4d), and evaluating the corresponding means and standard deviations for each age group.

Since particle size, airway geometry and flow rates all influence the Stokes number (Eq 1), any variable change with age will translate to a different aerosol size at a fixed Stk. As discussed above, our results (Fig 4c and 4d) point to the existence of a single dimensionless “master curve” for conductive airway deposition, with a discernible maximum for each inhalation maneuver. This outcome offers an optimal particle size range that is indeed age-dependent. Following the results of Fig 4, we extract this optimal aerosol size range for targeted delivery under DPI (Fig 4c) and nebulizer inhalation (Fig 4d), respectively, for each age point. The bar plots of Fig 6 summarize the optimal aerosol sizes; particle diameters are evaluated by fitting Gaussian curves to the deposition efficiencies for each inhalation maneuver (Fig 4c and 4d) and extracting *d*_*p*_ from Eq (1) at each age point. Following an ANOVA test run on the individual curves of particle deposition efficiency as a function of particle diameter for all six cases (three age points for each DPI and nebulizer inhalation), we find statistical significance between results for DPI and nebulizer cases (p < 0.05). Furthermore, within the DPI inhalation cases the 5 and 25 year old cases exhibit the strongest differences (p < 0.05). We recall that since all the dimensional curves for deposition efficiency (Fig 4c and 4d) collapse onto a single non-dimensional curve as a function of Stokes number, our results emphasize importantly a path to extract the appropriate particle diameter range corresponding to the ‘optimal’ Stokes number for maximum deposition efficiency.

In general, our findings highlight how smaller particle sizes correlate with better deposition outcomes at a younger age. In particular in the DPI case, the most efficient particle sizes to deposit in the conductive upper airways (i.e. ∼5-6 *μ*m) are noticeably smaller when compared to the nebulizer case (∼12-15 *μ*m). This can be better understood if we recall that flow rates involved in DPI inhalation are significantly higher compared to tidal breathing during nebulizer inhalation. In turn, aerosols with larger diameters deposit primarily in the mouth-throat region due to impaction (i.e. larger Stk values), as seen in Fig 5, whereas smaller aerosols can lodge more distally into the conducting airways.

To date, inhalation therapies are typically inclined towards formulating pediatric dosages based on body weight [29, 30], with most aerosol deposition studies focused on adults. Our *in silico* study suggests that the fate of inhaled aerosols in the upper airways is well captured, to a first approximation, from the dimensionless curves of Fig 4. These can then be translated to extract age-specific aerosol sizes for optimizing upper airway delivery (Fig 6). With the existence of deposition optima as a function of Stk, our results hence advocate that designing inhaled therapeutics for optimized pediatric deposition outcomes would take into account the aerosol size and inhalation maneuver dependence. While we shed light on this specific aerosol transport question and identifying plausible deposition outcomes based on size and age, our results do not directly address evaluating the dosage requirements that would potentially be best suited for an inhalation therapy if the “optimized” size distributions were indeed available for each age group.

The present study is limited by its idealized nature but serves as a first step in bringing forward aerodynamic determinants in selecting aerosol sizes for targeted delivery in pediatric upper airways. Indeed, subject-specific differences in airway geometry and breathing profiles [25], even at a given age point, are additional parameters that undeniably influence deposition outcomes. Moreover, factors such as anatomical abnormalities (e.g. airway constrictions, dysanapsis) that lead to ventilation inhomogeneity [93] will affect deposition outcomes. Finally, our discussion omits the fate of aerosols in more distal bronchi, namely those particles transiting beyond the (seven) upper generations of the present model. Despite such limitations, the present study adds voice to the growing needs of shifting current guidelines for pediatric patients to more realistic age-based drug delivery systems.

## Conclusions

In the present work we have drawn on *in silico* CFD methods using an idealized, anatomically-faithful airway geometry to simulate airflow and inhaled aerosol transport spanning a five year old to an adult. Breathing conditions were chosen to mimic realistic age-specific inhalation maneuvers representative of DPI and nebulizer inhalation. Simulation results underline the overwhelming similarities in flow topologies across age points. Interestingly, we uncover that deposition efficiency is captured by a single curve governed via the non-dimensional Stokes number (Stk) for each inhalation maneuver. In particular in the conductive airways, such curves are characterized by the existence of a distinct deposition peak irrespective of age. For the DPI simulations, this peak (about 80%) occurs at Stk ≈ 0.06 whereas for nebulizer simulations, the corresponding peak (45%) occurs in the range of Stk between 0.03-0.04. In the context of pediatric inhalation therapy, such dimensionless findings translate to the existence of an optimal aerosol size range that thus evolves with age and varies according to inhalation device. While implications of our results for current particle manufacturing techniques lie beyond the scope of the present work (e.g. adapting particle sizing via spray drying techniques for DPI powders or changing mesh sizes in nebulizer devices), the role of aerodynamic determinants in determining the fate of inhaled aerosols suggests a fresh outlook on drug delivery systems for pediatric inhalation therapies.

## Supporting information

S1 Fig Velocity and turbulent intensity contours for nebulizer simulations. Contour maps of (a) velocity magnitude (|**u**|) and (b) turbulent intensity at peak inhalation for nebulizer simulations (Fig 1c). Contour maps are rendered across the center plane cutting through the mouth-throat and trachea; the 2D center plane is schematically shown in the sliced section of the 3D CAD geometry on the left. Maps are shown across the three age points of 5, 10 and 25 years, corresponding to geometries of increasing size in (a) and (b), respectively.
(PDF)

S1 File Airway geometry. The idealized conducting airway geometry which includes seven generations of airways is made available for further use.
(STL)

S1 Video Aerosol motion. The video shows aerosol inhalation through a DPI maneuver in an idealized airway geometry representing a 5 year old child. The aerosols introduced range between 1 *μ*m to 12 *μ*m.
(MP4)

S2 Video Aerosol deposition. The video shows aerosol deposition as a function of time for a DPI maneuver in an idealized airway geometry representing a 5 year old child. The aerosols introduced range between 1 *μ*m to 12 *μ*m. Aerosols larger than 6 *μ*m are colored red and size of the aerosols are scaled according to particle diameter.
(MP4)
