# Supplementary material for: Targeting inhaled aerosol delivery to upper airways in children: Insight from computational fluid dynamics (CFD)
Source: PLoS One. 2018 Nov 20;13(11):e0207711. doi: 10.1371/journal.pone.0207711 (PMC6245749; doi:10.1371/journal.pone.0207711)
Supplement: S1 Fig — Contour maps of (a) velocity magnitude (|u|) and (b) turbulent intensity at peak inhalation for nebulizer simulations (Fig 1c). Contour maps are rendered across the center plane cutting through the mouth-throat and trachea; the 2D center plane is schematically shown in the sliced section of the 3D CAD geometry on the left. Maps are shown across the three age points of 5, 10 and 25 years, corresponding to geometries of increasing size in (a) and (b), respectively. (PDF) [file pone.0207711.s001.pdf]

S1 Fig. Velocity and turbulent intensity contours for nebulizer simulations.

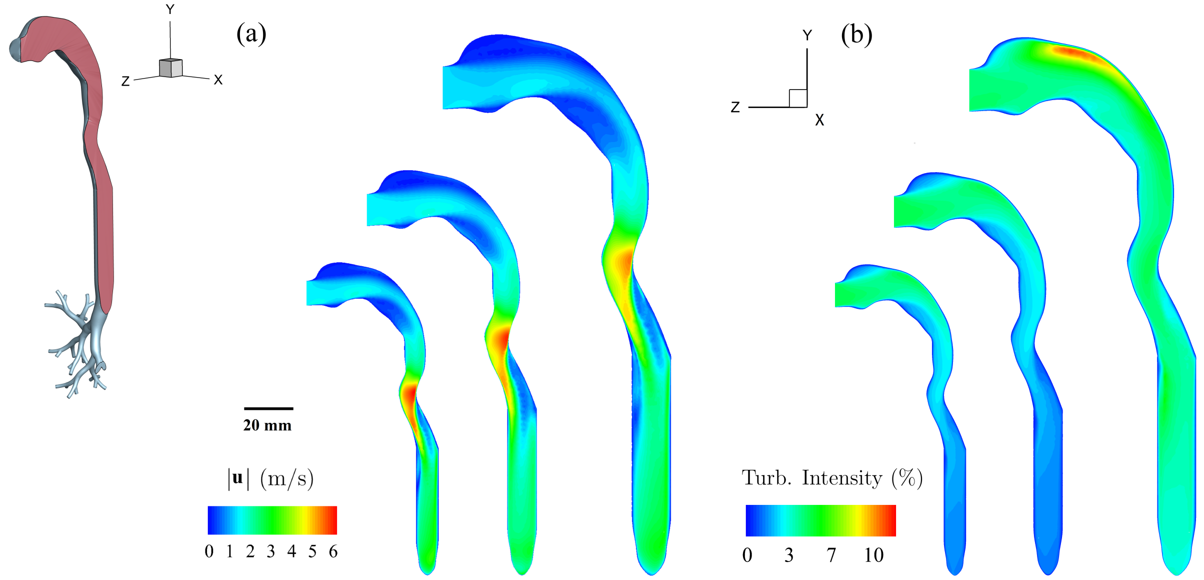

Contour maps of (a) velocity magnitude ($|\mathbf{u}|$) and (b) turbulent intensity at peak inhalation for nebulizer simulations (Fig.1c). Contour maps are rendered across the center plane cutting through the mouth-throat and trachea; the 2D center plane is schematically shown in the sliced section of the 3D CAD geometry on the left. Maps are shown across the three age points of 5, 10 and 25 years, corresponding to geometries of increasing size in (a) and (b), respectively.
